# Supplementary material for: Checkpoint kinase 1 is essential for fetal and adult hematopoiesis
Source: EMBO Rep. 2019 Jun 17;20(8):e47026. doi: 10.15252/embr.201847026 (PMC6680171; doi:10.15252/embr.201847026)
Supplement: Supplementary file 1 — Appendix [file EMBR-20-e47026-s001.pdf]

APPENDIX

Checkpoint kinase-1 is essential for fetal and adult hematopoiesis

Fabian Schuler[1], Sehar Afreen[2,3], Claudia Manzl[4], Georg Häcker[5], Miriam Erlacher[2,6] and Andreas Villunger[1,7,8]

[1]Division of Developmental Immunology, Biocenter, Medical University of Innsbruck, Innsbruck, Austria. [2]Division of Pediatric Hematology and Oncology, Department of Pediatrics and Adolescent Medicine, Faculty of Medicine, University of Freiburg, Freiburg, Germany. [3]Faculty of Biology, University of Freiburg, Freiburg, Germany. [4]Institute of Pathology, Neuropathology and Molecular pathology, Medical University of Innsbruck, Innsbruck, Austria. [5]Institute of Medical Microbiology and Hygiene, University Medical Center Freiburg, Freiburg, Germany. [6]German Cancer Consortium (DKTK), Freiburg, Germany and German Cancer Research Center (DKFZ), Heidelberg, Germany. [7] CeMM Research Center for Molecular Medicine of the Austrian Academy of Sciences, Vienna 1090, Austria. [8] Ludwig Boltzmann Institute for Rare and Undiagnosed Diseases, Vienna 1090, Austria

Table of Contents

Appendix Figure S1: CHK1-inhibition kills primary murine hematopoietic stem and progenitor (HSPC) cells. page 2

Appendix Figure S2: Tracing acute depletion of Chk1 in the hematopoietic system based on GFP-reporter expression page 3

Appendix Figure S3: Tracing acute depletion of Chk1 in the hematopoietic system based on GFP-reporter expression page 4

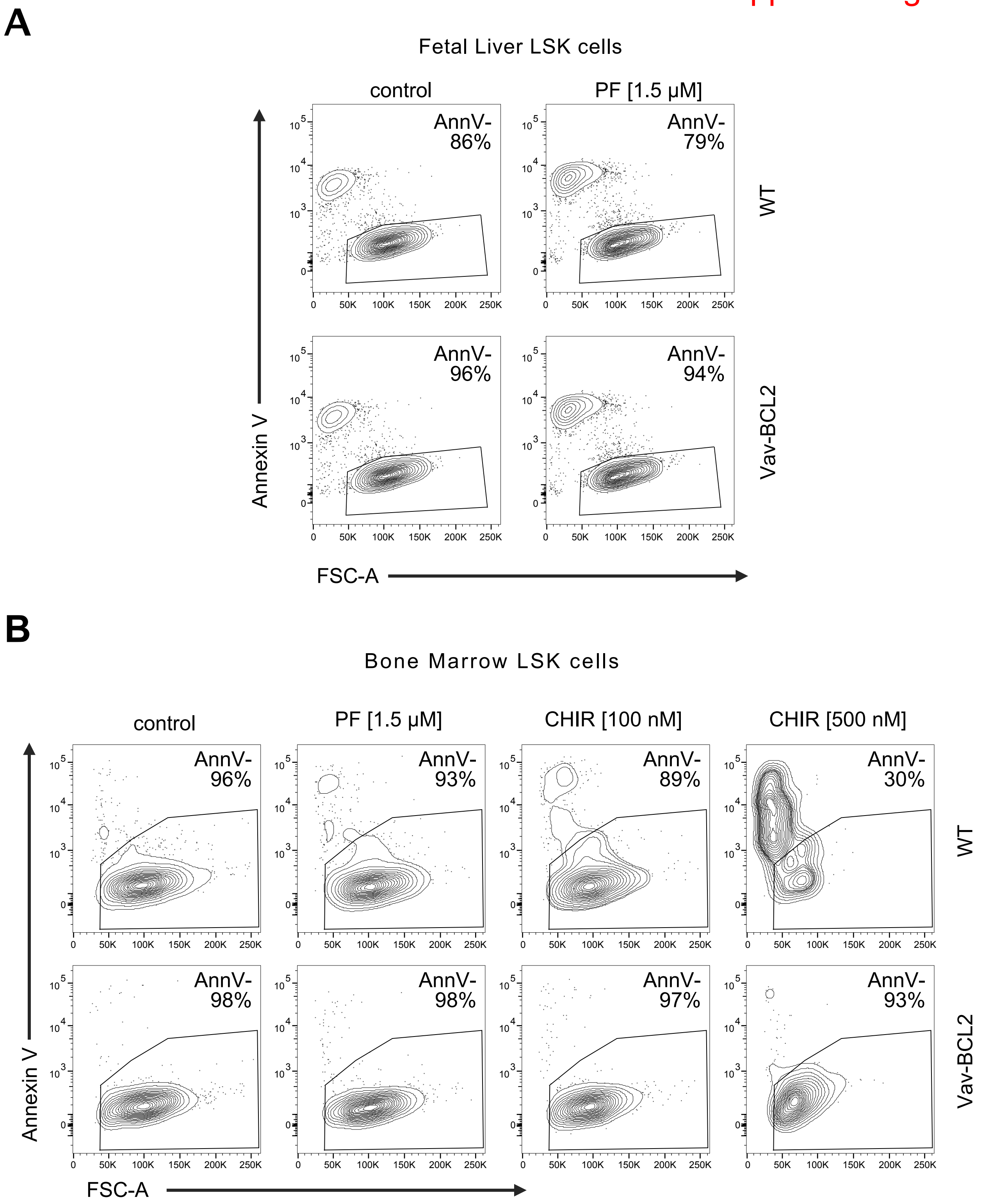

Appendix Figure S1: CHK1-inhibition kills primary murine hematopoietic stem and progenitor (HSPC) cells.

(A, B) Gating strategy (for Figure 2A) used to determine cell death (Annexin V) in response to CHK1i of sorted Fetal liver-LSK **(A)** and Bone Marrow LSK **(B)** cells.

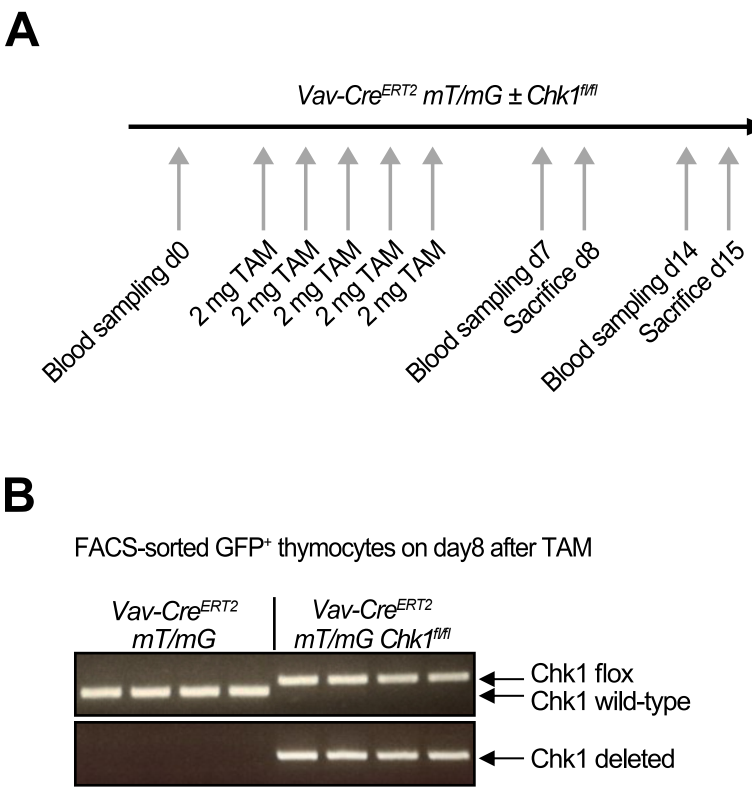

Appendix Figure S2: Tracing acute depletion of Chk1 in the hematopoietic system based on GFP-reporter expression

(A) Scheme of tamoxifen (TAM) administration by gavage (5 x 2 mg in 200 µl/mouse, daily), blood or tissue sampling times using *Chk1*$^{fl/fl}$ *VavCRE*^{ERT2} *mT/mG* (*Chk1*$^{fl/fl}$) or *VavCRE*^{ERT2} *mT/mG* (control) mice.

(B) PCR analysis amplifying the Chk1 wt, flox and deleted allele in FACS-sorted GFP+ thymocytes of individual mice on day 8 after the first administration of tamoxifen.

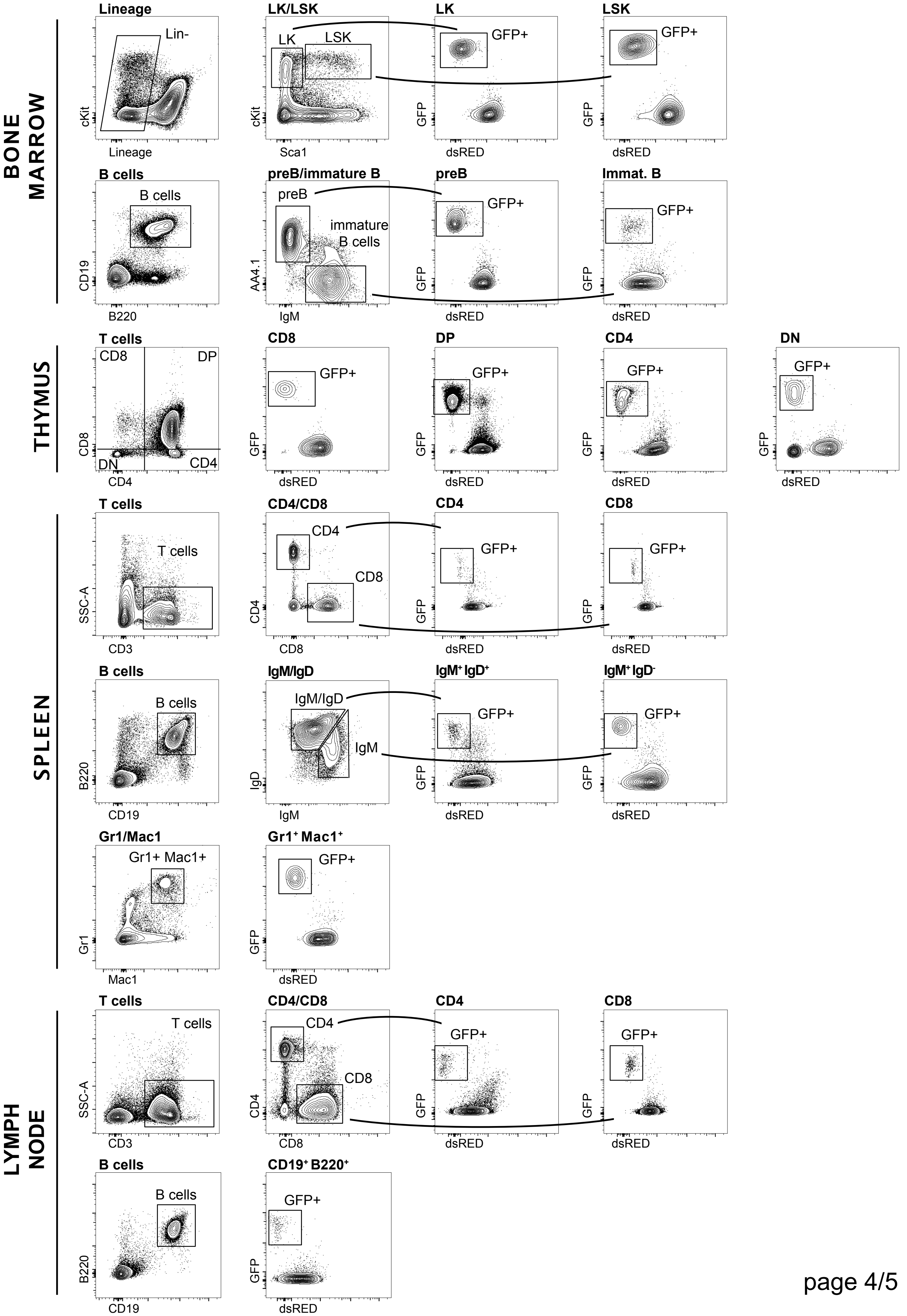
BONE MARROW
Lineage
Lin-
cKit
Lineage
LK/LSK
LK
LSK
cKit
Sca1
LK
GFP+
GFP
dsRED
LSK
GFP+
GFP
dsRED
B cells
B cells
CD19
B220
preB/immature B
preB
immature B cells
AA4.1
IgM
preB
GFP+
GFP
dsRED
Immat. B
GFP+
GFP
dsRED
THYMUS
T cells
CD8
DP
DN
CD4
CD8
CD4
CD8
GFP+
GFP
dsRED
DP
GFP+
GFP
dsRED
CD4
GFP+
GFP
dsRED
DN
GFP+
GFP
dsRED
SPLEEN
T cells
T cells
SSC-A
CD3
CD4/CD8
CD4
CD8
CD4
CD8
CD4
GFP+
GFP
dsRED
CD8
GFP+
GFP
dsRED
B cells
B cells
B220
CD19
IgM/IgD
IgM/IgD
IgM
IgD
IgM
IgM+ IgD+
GFP+
GFP
dsRED
IgM+ IgD-
GFP+
GFP
dsRED
Gr1/Mac1
Gr1+ Mac1+
Gr1
Mac1
Gr1+ Mac1+
GFP+
GFP
dsRED
LYMPH NODE
T cells
T cells
SSC-A
CD3
CD4/CD8
CD4
CD8
CD4
CD8
CD4
GFP+
GFP
dsRED
CD8
GFP+
GFP
dsRED
B cells
B cells
B220
CD19
CD19+ B220+
GFP+
GFP
dsRED

Appendix Figure S3: Tracing acute depletion of Chk1 in the hematopoietic system based on GFP-reporter expression

Gating strategy for the quantification of different hematopoietic cell subsets in *Vav-Cre*^{ERT2} *mT/mG* (ctrl) and *Vav-Cre*^{ERT2} *mT/mG Chk1*$^{fl/fl}$ (flox) mice 8 or 15 days after the first administration of tamoxifen, shown in Figure 8 and in Table EV1.
