# Supplementary material for: Checkpoint kinase 1 is essential for fetal and adult hematopoiesis
Source: EMBO Rep. 2019 Jun 17;20(8):e47026. doi: 10.15252/embr.201847026 (PMC6680171; doi:10.15252/embr.201847026)
Supplement: Supplementary file 2 — Expanded View Figures PDF [file EMBR-20-e47026-s002.pdf]

Expanded View Figures

Figure EV1. Inhibition of CHK1 results in BCL2-regulated cell death involving BIM in MPP-like Hoxb8-FL cells.

A Quantification and significant differences in the subG1 (left) and G1 fraction (right) of Hoxb8-FL cells of the indicated genotypes treated with CHK1i. The corresponding analysis of cell cycle distribution (subG1, G1, S, G2, polyploid) is shown in Fig 1A.

B Hoxb8-FL cells of the indicated genotypes were treated for 24 h with the inhibitors PF-477736 (PF) or CHIR-124 (CHIR) and analyzed by Nicoletti staining.

C Quantification of subG1 cells as a surrogate for cell death induced by CHK1i in Hoxb8-FL cells of the indicated genotypes.

Data information: Bars represent means ± s.e.m. (n = 3/per genotype). Asterisks indicate significant differences compared to control (DMSO): *$P < 0.05$, **$P < 0.01$, ***$P < 0.001$ using unpaired Student's t-test.

© 2019 The Authors

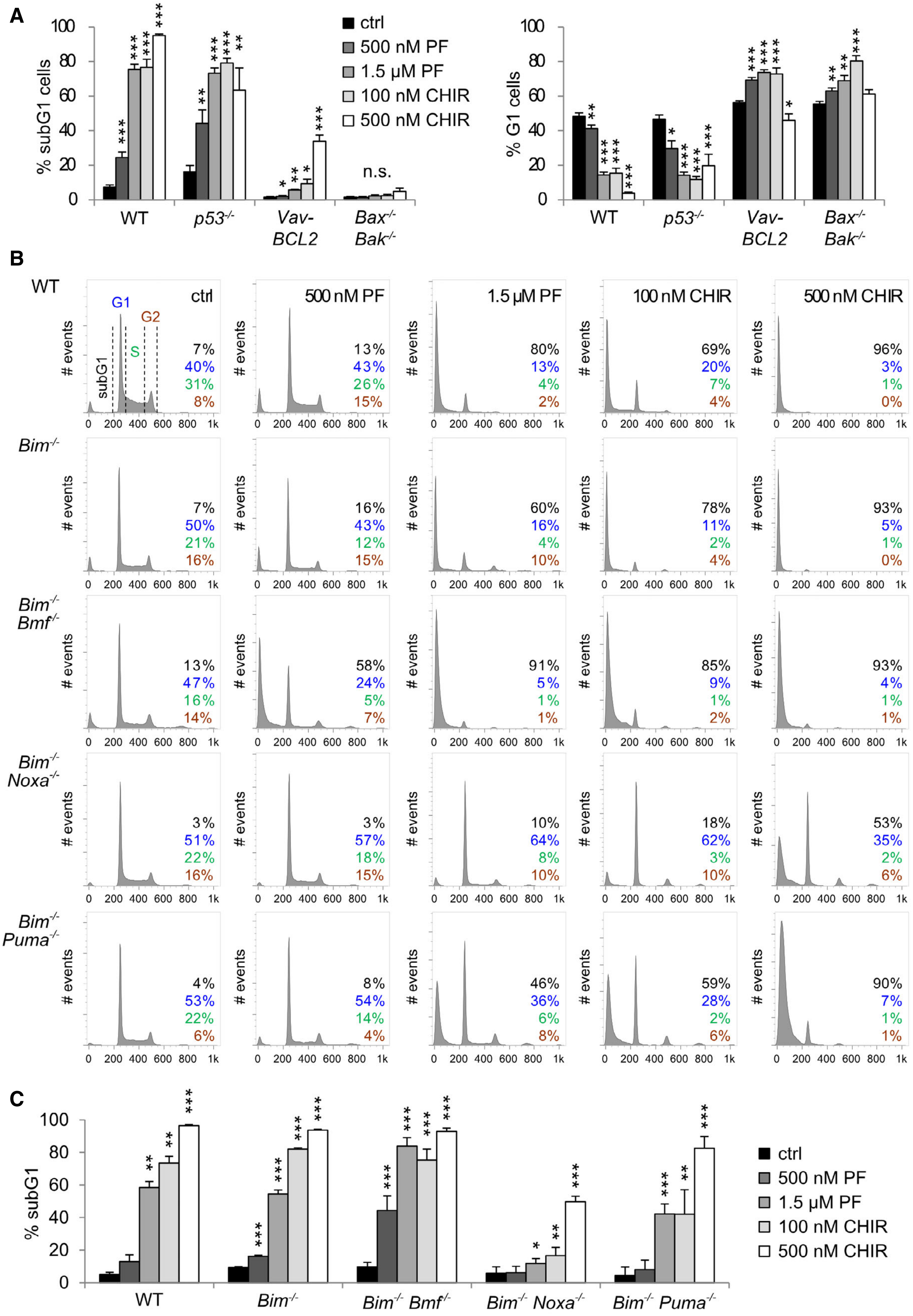

Figure EV1.

 © 2019 The Authors

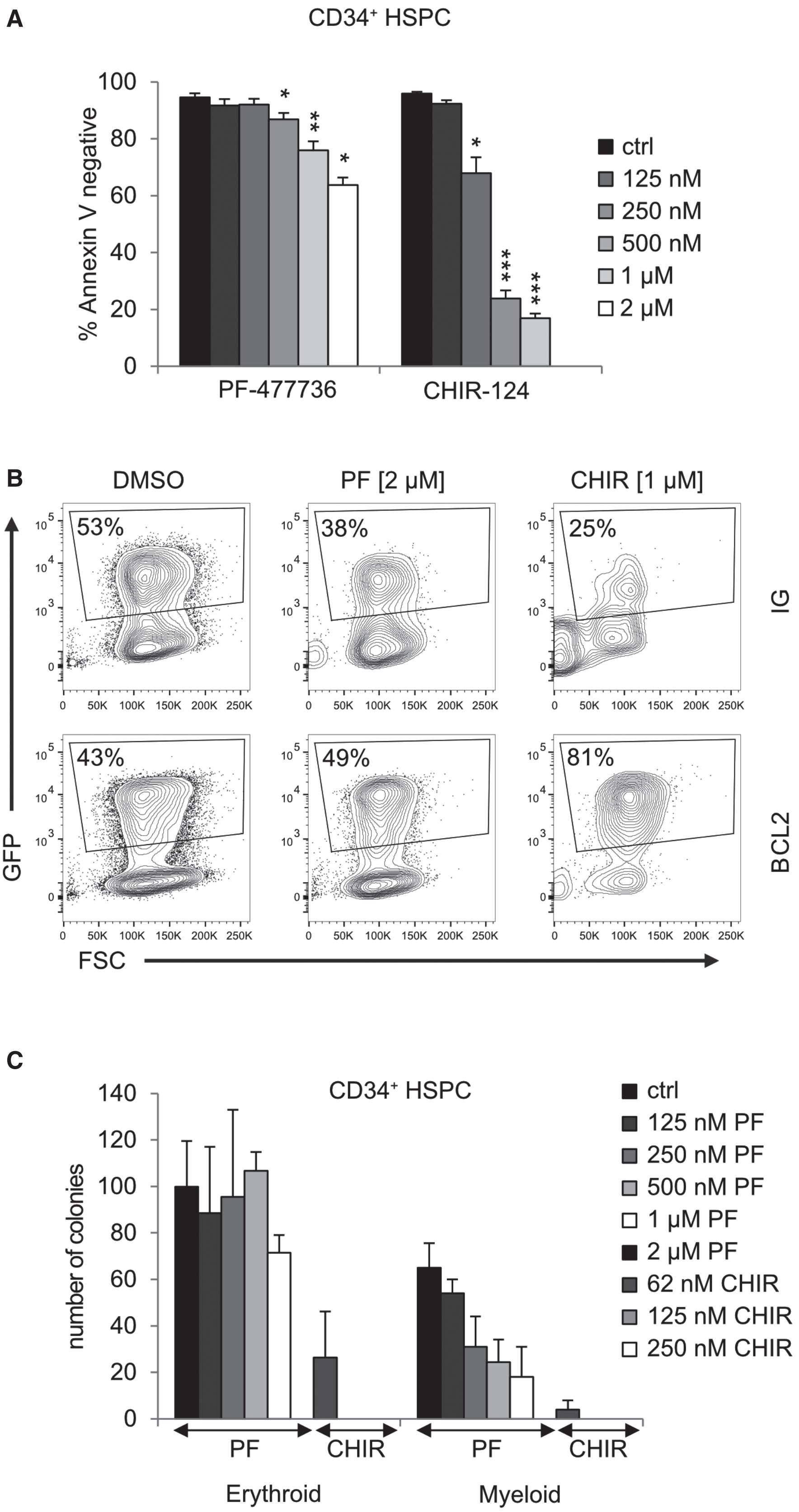

Figure EV2. Human CD34+ HSPCs undergo a BCL2-regulated apoptotic cell death in response to CHK1 inhibition.

A MACS-purified CD34+ human cord blood-derived HSPCs were treated for 48 h with graded doses of PF-477736 or CHIR-124. Cell death was assessed using Annexin V/7-AAD staining and flow cytometry. N = 4/genotype.

B Related to Fig 3C: CD34+ cells were transduced with a lentiviral empty vector (IG) or a vector encoding BCL2, along with IRES-GFP, followed by CHK1i application for 48 h. Shown here are the percentages of GFP+ cells within living cells.

C CD34+ HSPCs was assessed for their colony formation capacity in methyl cellulose assays in the presence of graded doses of CHK1i. Colonies were counted 10 days postseeding of 1,000 CD34+ cells. "Myeloid colonies" include granulocyte/monocyte, monocyte, granulocyte, and GEMM (granulocyte, erythroid, monocyte/macrophage, and megakaryocyte) colonies. N = 3–6 donors.

Data information: Bars represent means ± s.e.m. Asterisks indicate significant differences: *$P < 0.05$, **$P < 0.01$, ***$P < 0.001$ using unpaired Student's t-test.

Figure EV3. Deletion of *Chk1* in HSC prevents fetal erythropoiesis.

A Related to Fig 4E: Representative dot-plots used to quantify different erythroid cell populations in the fetal liver at E13.5 of the different genotypes.
B Quantification of the relative distribution of the cell population shown in (A). Large "EryA" erythroblasts ($CD71^{high}$ $Ter119^{high}$ FSC^{high}), smaller, more mature "EryB" erythroblasts ($CD71^{high}$ $Ter119^{high}$ FSC^{low}); most mature erythroblast subset is EryC ($CD71^{low}$ $Ter119^{high}$ FSC^{low}). Bars represent means ± s.e.m. ctrl $N = 5$ ($N = 4$ $Chk1^{fl/+}$ and $N = 1$ $Chk1^{fl/+}$ *Vav-Cre*), $N = 3$ for $Chk1^{fl/-}$ *Vav-Cre*. Asterisks indicate significant differences: *$P < 0.05$, **$P < 0.01$, ***$P < 0.001$ using unpaired Student's *t*-test.

 © 2019 The Authors

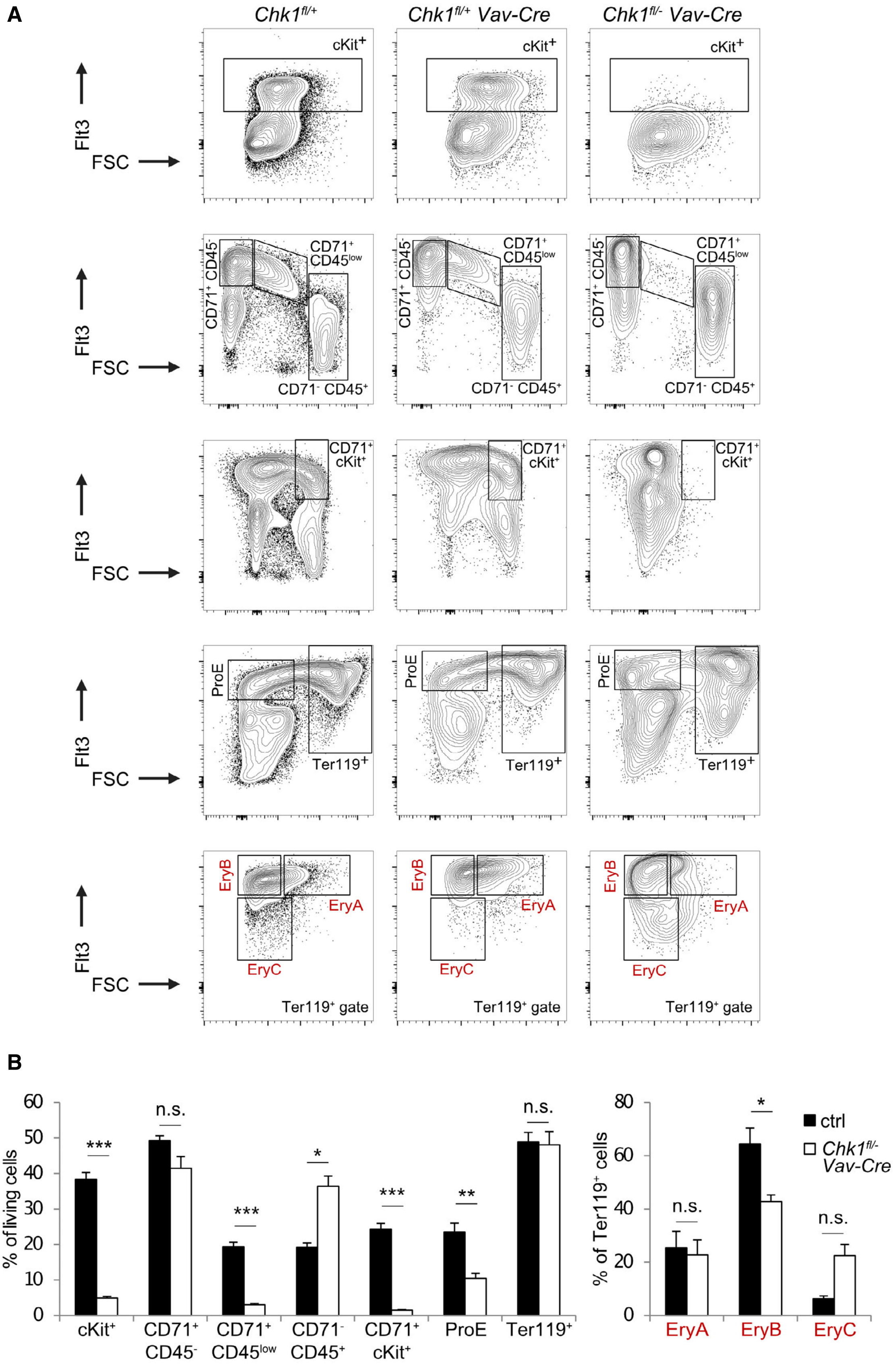
A
Chk1fl/+
Chk1fl/+ Vav-Cre
Chk1fl/- Vav-Cre
cKit+
CD71+ CD45-
CD71+ CD45low
CD71- CD45+
CD71+ cKit+
ProE
Ter119+
EryA
EryB
EryC
Ter119+ gate
Flt3
FSC
B
% of living cells
% of Ter119+ cells
ctrl
Chk1fl/- Vav-Cre
n.s.
***
**
*
cKit+
CD71+ CD45-
CD71+ CD45low
CD71- CD45+
CD71+ cKit+
ProE
Ter119+
EryA
EryB
EryC
0
10
20
30
40
50
60
80

Figure EV3.

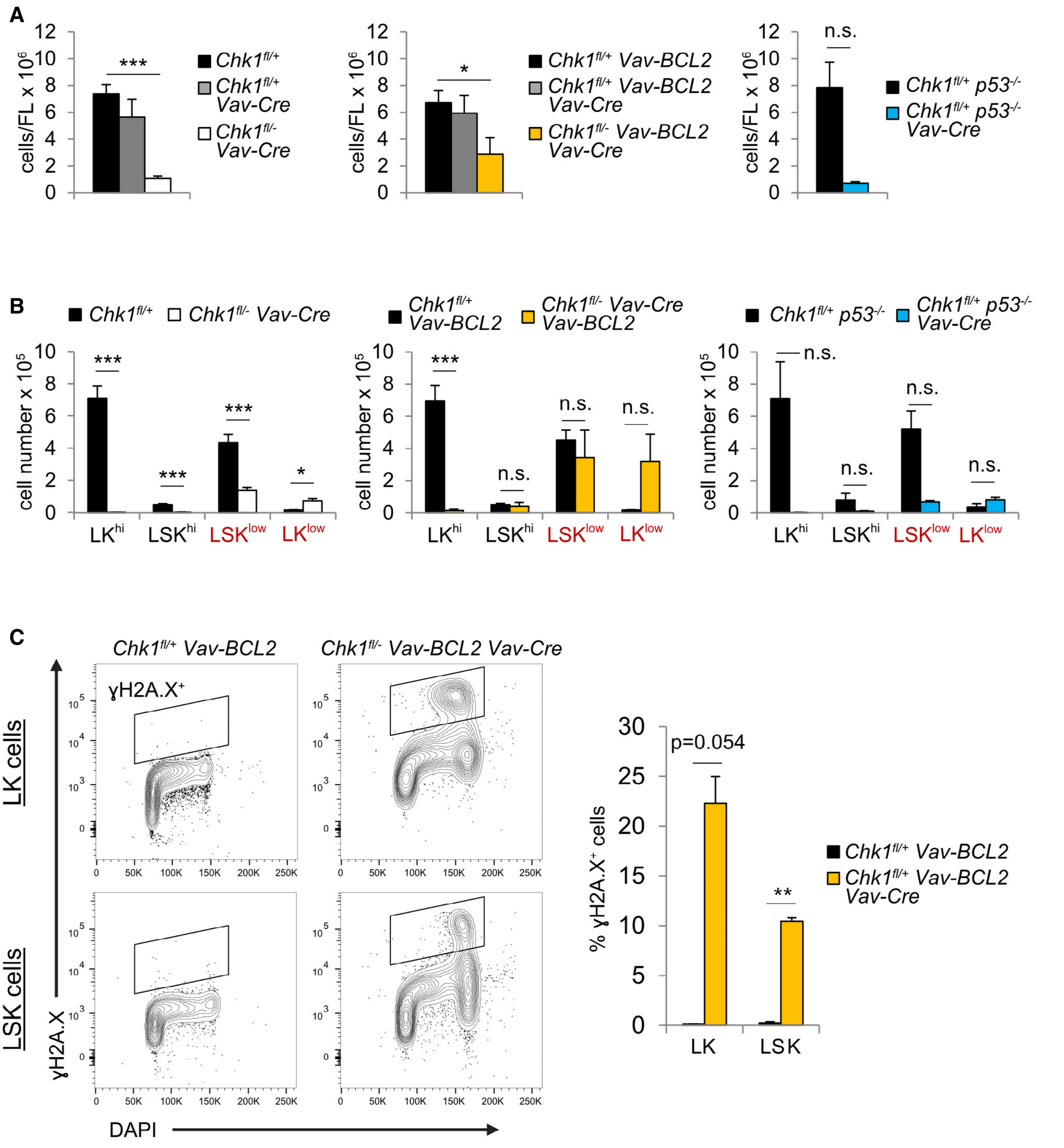

Figure EV4. BCL2 overexpression does not prevent DNA damage nor stem cell loss upon CHK1 deletion.

A Quantification of the total cell number found in the E13.5 fetal liver of the indicated genotypes.

B Calculation of the LK^{hi}/LK^{low} and LSK^{hi}/LSK^{low}-cell number per fetal liver at E13.5 of the indicated genotypes.

C LK and LSK cells of the indicated genotypes were sorted from E13.5 fetal livers and immediately fixed in 70% EtOH. Fixed cell suspensions were stained for γH2A.X and DAPI intracellularly. Shown here are representative dot-plots (left) and quantification (right) of $N = 2$ animals per genotype; means ± s.d.

Data information: For (A) and (B): *Chk1*$^{fl/+}$ ($n = 21$ embryos from 5 litters, 21/5), *Chk1*$^{fl/+}$ *Vav-Cre* ($n = 5/5$), *Chk1*$^{fl/-}$ *Vav-Cre* ($n = 5/3$), *Chk1*$^{fl/+}$ *Vav-BCL2* ($n = 16/5$), *Chk1*$^{fl/+}$ *Vav-Cre Vav-BCL2* ($n = 7/3$), *Chk1*$^{fl/-}$ *Vav-Cre Vav-BCL2* ($n = 5/3$), *Chk1*$^{fl/+}$ *p53*$^{-/-}$ ($n = 2/1$), and *Chk1*$^{fl/-}$ *Vav-Cre p53*$^{-/-}$ ($n = 4/2$). Data information for (A–C): Asterisks indicate significant differences: $^{*}P < 0.05$, $^{**}P < 0.01$, $^{***}P < 0.001$ using unpaired Student's *t*-test.

© 2019 The Authors

Figure EV5. Blood cell analysis after tamoxifen administration.

Quantification of cellular parameters of peripheral blood cells sampled from *Vav-Cre*^{ERT2} *mT/mG* (ctrl) and *Vav-Cre*^{ERT2} *mT/mG Chk1*$^{fl/fl}$ (flox) mice on day 7 or day 14 after the first administration of tamoxifen. Values represent means ± s.e.m. of two independent experimental cohorts (Day 0 $N = 12$ ctrl, $N = 11$ flox, Day 7 $N = 11$ ctrl, $N = 14$ flox, Day 14 $N = 4$ ctrl, $N = 8$ flox). Two-way ANOVA analysis revealed no significant differences between the two experimental groups in either of the parameters analyzed. Abbreviations: WBC (10^3/mm^3), white blood cells; RBC (10^6/mm^3), red blood cells; HGB (g/dl), hemoglobin; HCT (%), hematocrit; PLT (10^3/mm^3), platelets; %Lymph, percentage of lymphocytes; %MO, percentage of monocytes; %GRA, percentage of granulocytes; MCV (μm^3), mean corpuscular volume; MCH (pg), mean corpuscular hemoglobin; MCHC (g/dl), mean corpuscular hemoglobin concentration; RDW [%], red cell distribution width; MPV (μm^3), mean platelet volume; #LYM (10^3/mm^3), number of lymphocytes; #MO (10^3/mm^3), number of monocytes; #GRA (10^3/mm^3), number of granulocytes.

© 2019 The Authors

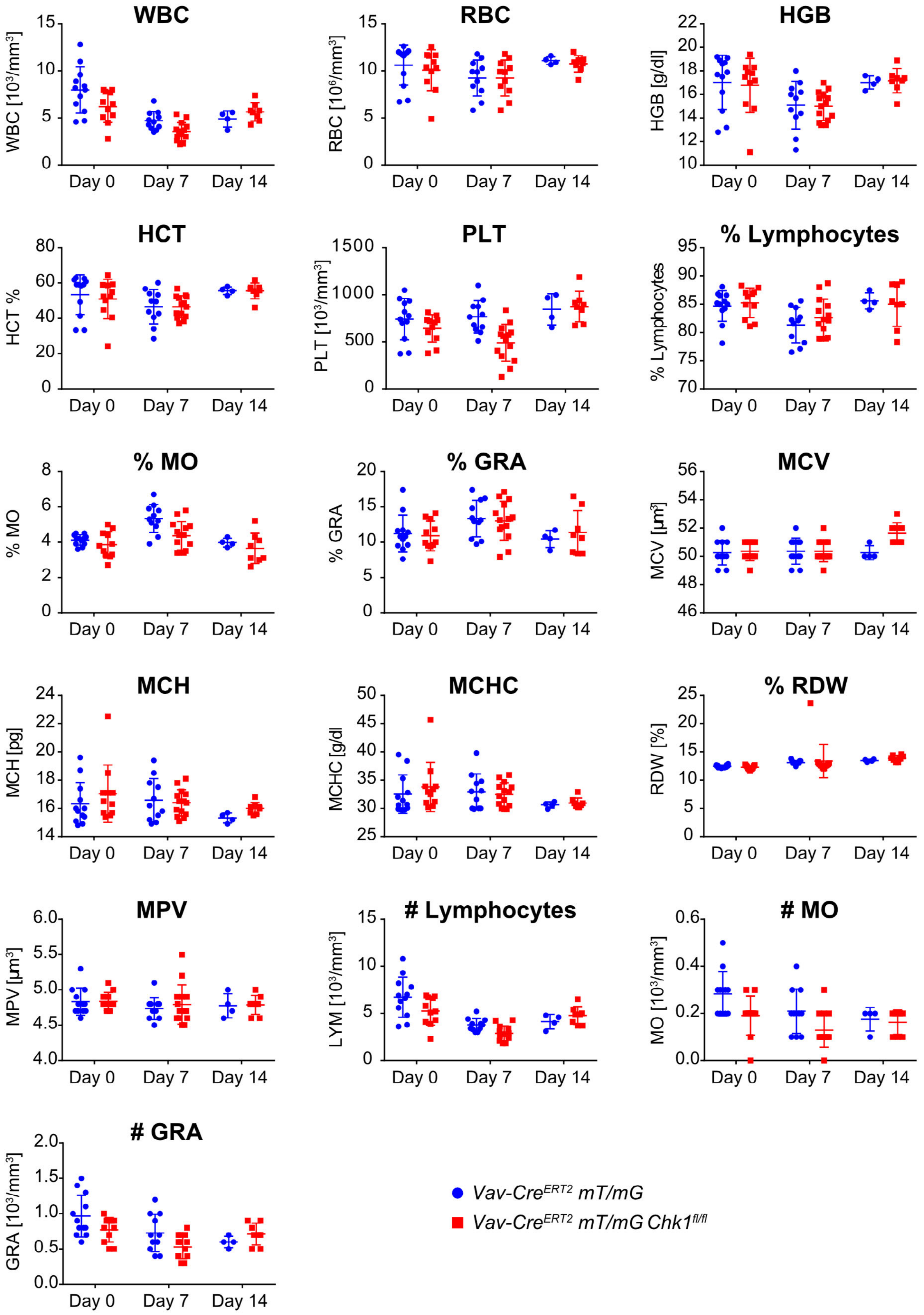

Figure EV5.

© 2019 The Authors
