# Supplementary figures and images for: Checkpoint kinase 1 is essential for fetal and adult hematopoiesis
Source: EMBO Rep. 2019 Jun 17;20(8):e47026. doi: 10.15252/embr.201847026 (PMC6680171; doi:10.15252/embr.201847026)

Uncropped Western Blots

Schuler et al\_Fig. 1C

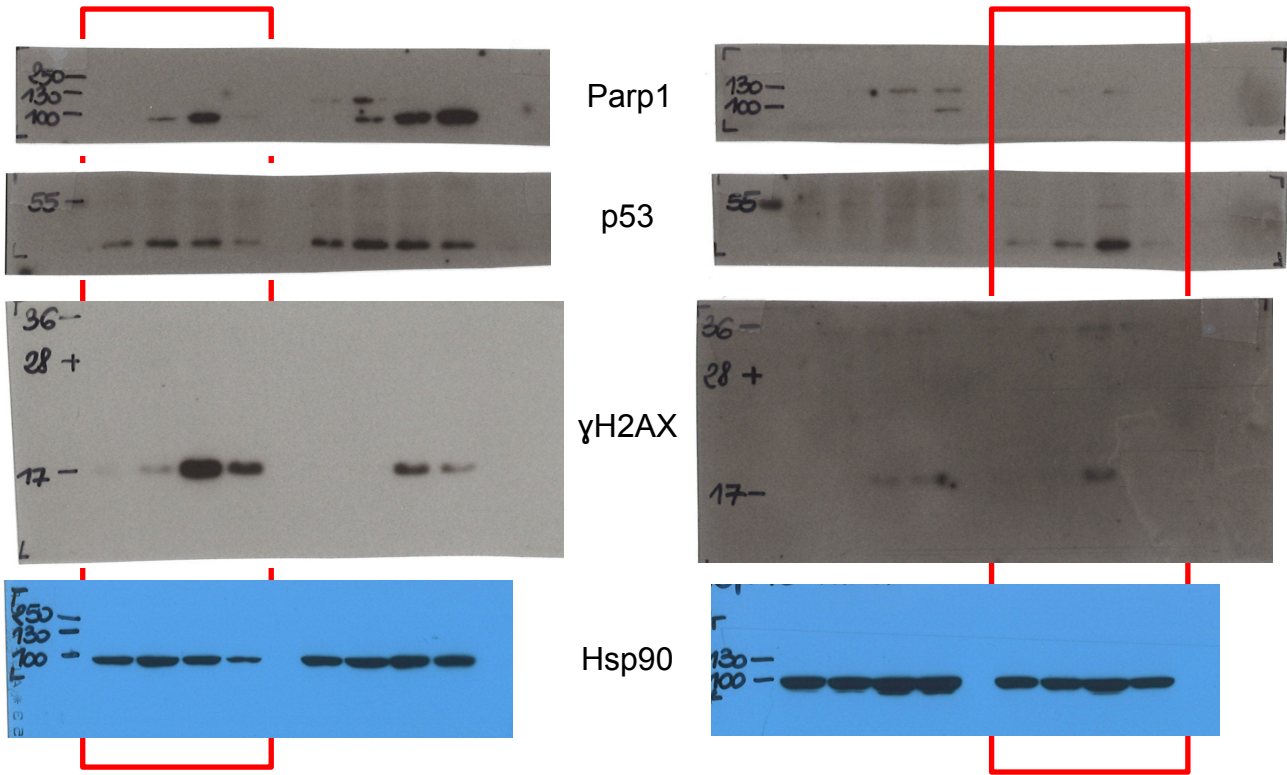

Supplement: Supplementary file 5 — Source Data for Figure 1 [file EMBR-20-e47026-s004.pdf]

Schuler et al\_Fig. 6B

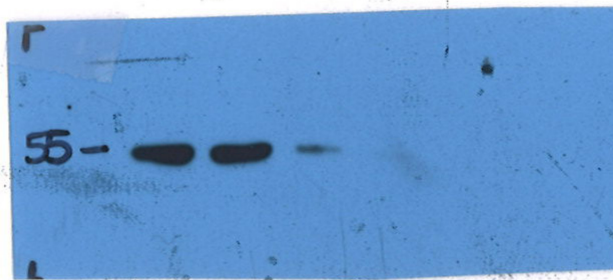

Chk1

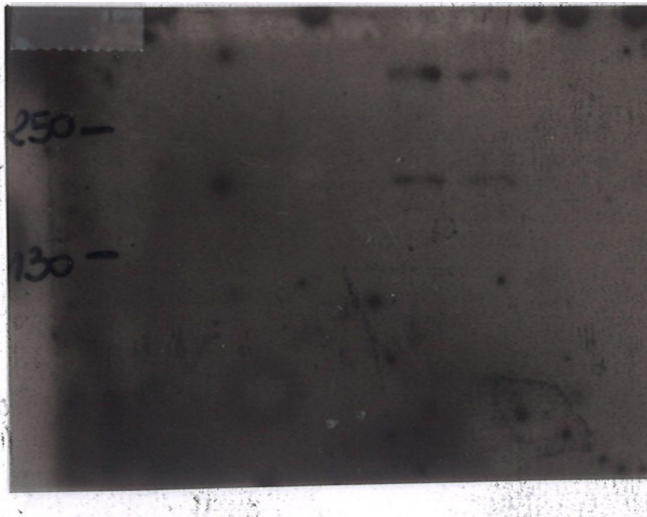

pATR-Ser428

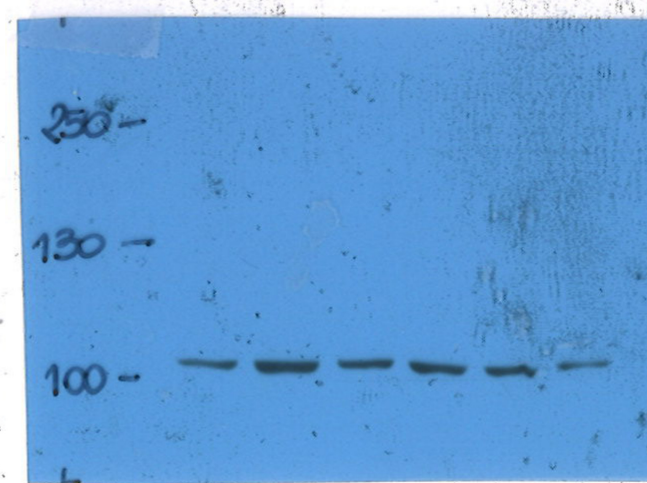

Hsp90

Supplement: Supplementary file 6 — Source Data for Figure 6 [file EMBR-20-e47026-s005.pdf]

Schuler et al\_Fig. 7D

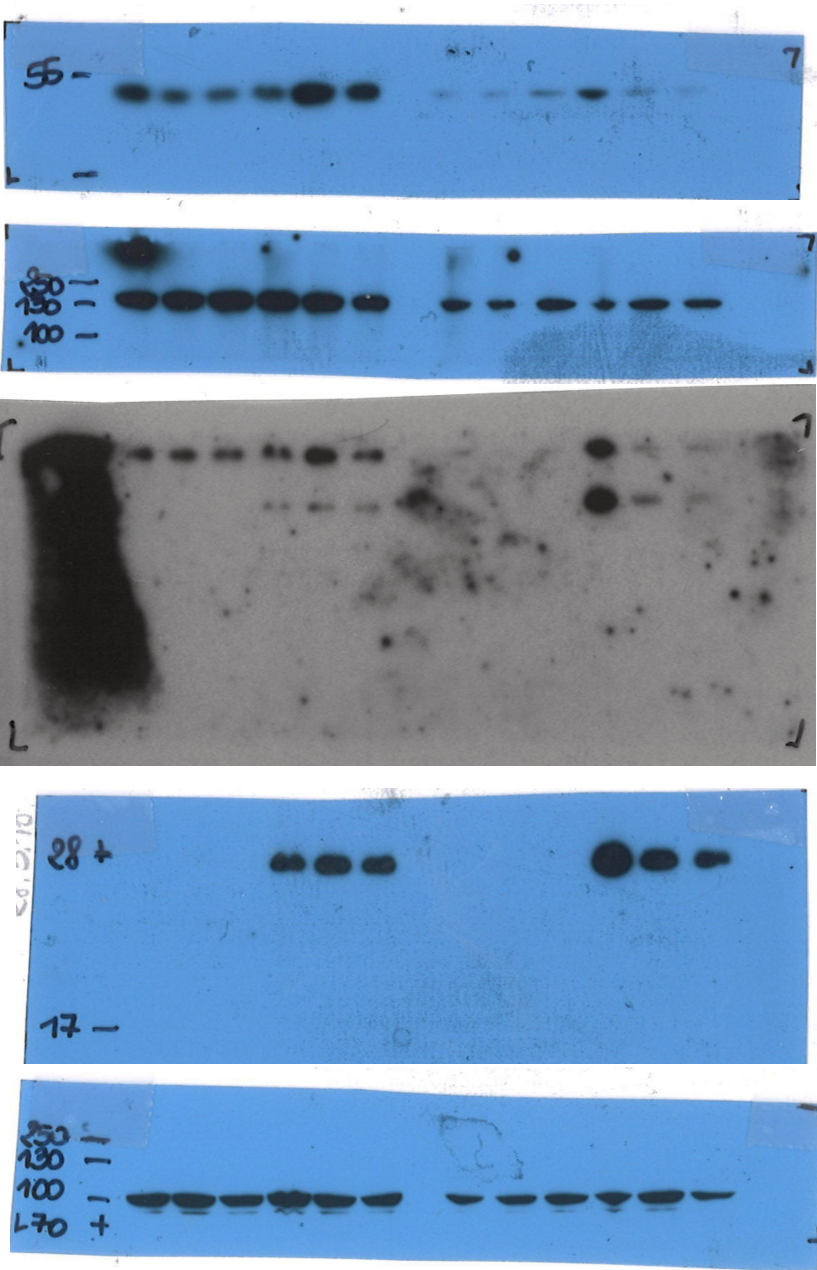

Chk1

Parp1

CycD3

hBCL2

Hsp90

Supplement: Supplementary file 7 — Source Data for Figure 7 [file EMBR-20-e47026-s006.pdf]
