# Supplementary material for: Checkpoint kinase 1 is essential for fetal and adult hematopoiesis
Source: EMBO Rep. 2019 Jun 17;20(8):e47026. doi: 10.15252/embr.201847026 (PMC6680171; doi:10.15252/embr.201847026)
Supplement: Supplementary file 8 — Source Data for Figure 8 [file EMBR-20-e47026-s007.pdf]

Uncropped Western Blots

Schuler et al_Fig. 8A

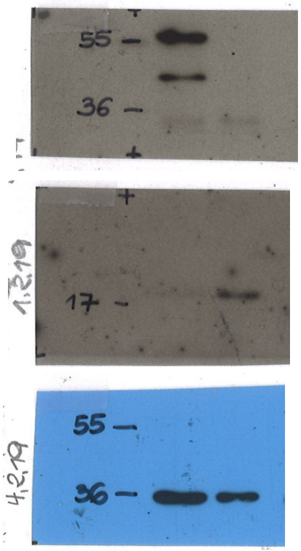

Chk1

γH2A.X

Gapdh

Schuler et al_Fig. 8B

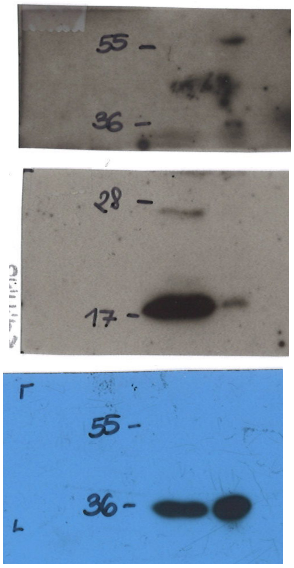

Chk1

γH2A.X

Western blot 8B has been „mirrored“ in the manuscript

Gapdh

Schuler et al_Fig. 8G

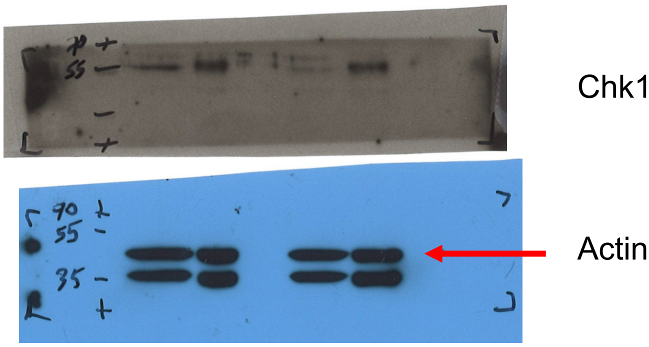

Chk1

Actin

Schuler et al_Fig. 8H

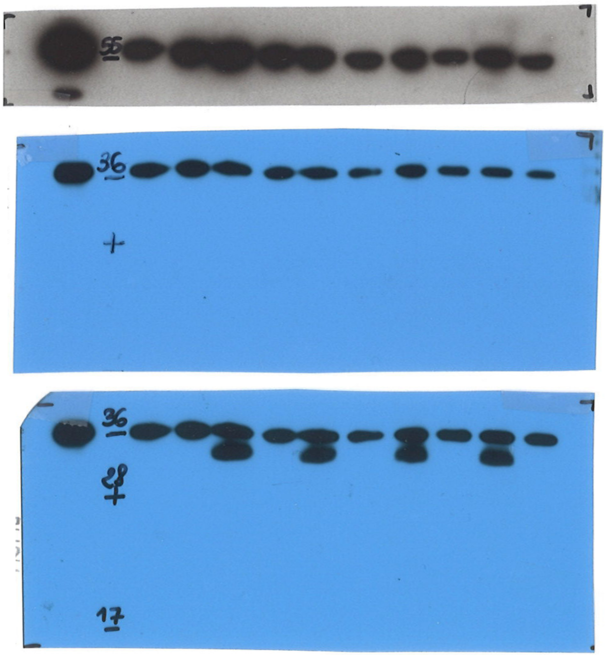

Chk1

Gapdh: not included in the paper

Gapdh
GFP (reprobed after GAPDH)
